# Multi-tiered external facilitation: the role of feedback loops and tailored interventions in supporting change in a stepped-wedge implementation trial

**DOI:** 10.1186/s43058-021-00180-3

**Published:** 2021-07-27

**Authors:** Lauren S. Penney, Teresa M. Damush, Nicholas A. Rattray, Edward J. Miech, Sean A. Baird, Barbara J. Homoya, Laura J. Myers, Dawn M. Bravata

**Affiliations:** 1 VA HSR&D Elizabeth Dole Center of Excellence for Veteran and Caregiver Research, South Texas Veterans Health Care System, San Antonio, TX USA; 2 School of Medicine, University of Texas Health at San Antonio, San Antonio, TX USA; 3 VA HSR&D Center for Health Information and Communication (CHIC), Richard L. Roudebush VA Medical Center, Indianapolis, IN USA; 4 Regenstrief Institute, Inc., Indianapolis, IN USA; 5 Department of Internal Medicine, Indiana University School of Medicine, Indianapolis, IN USA; 6 Department of Anthropology, Indiana University-Purdue University, Indianapolis, IN USA; 7 Department of Neurology, Indiana University School of Medicine, Indianapolis, IN USA

**Keywords:** Implementation science, Facilitation, Cerebrovascular disease, Veterans Health Administration

## Abstract

**Background:**

Facilitation is a complex, relational implementation strategy that guides change processes. Facilitators engage in multiple activities and tailor efforts to local contexts. How this work is coordinated and shared among multiple, external actors and the contextual factors that prompt and moderate facilitators to tailor activities have not been well-described.

**Methods:**

We conducted a mixed methods evaluation of a trial to improve the quality of transient ischemic attack care. Six sites in the Veterans Health Administration received external facilitation (EF) before and during a 1-year active implementation period. We examined how EF was employed and activated. Data analysis included prospective logs of facilitator correspondence with sites (160 site-directed episodes), stakeholder interviews (a total of 78 interviews, involving 42 unique individuals), and collaborative call debriefs (*n*=22) spanning implementation stages. Logs were descriptively analyzed across facilitators, sites, time periods, and activity types. Interview transcripts were coded for content related to EF and themes were identified. Debriefs were reviewed to identify instances of and utilization of EF during site critical junctures.

**Results:**

Multi-tiered EF was supported by two groups (site-facing quality improvement [QI] facilitators and the implementation support team) that were connected by feedback loops. Each site received an average of 24 episodes of site-directed EF; most of the EF was delivered by the QI nurse. For each site, site-directed EF frequently involved networking (45%), preparation and planning (44%), process monitoring (44%), and/or education (36%). EF less commonly involved audit and feedback (20%), brainstorming solutions (16%), and/or stakeholder engagement (5%). However, site-directed EF varied widely across sites and time periods in terms of these facilitation types. Site participants recognized the responsiveness of the QI nurse and valued her problem-solving, feedback, and accountability support. External facilitators used monitoring and dialogue to intervene by facilitating redirection during challenging periods of uncertainty about project direction and feasibility for sites. External facilitators, in collaboration with the implementation support team, successfully used strategies tailored to diverse local contexts, including networking, providing data, and brainstorming solutions.

**Conclusions:**

Multi-tiered facilitation capitalizing on emergent feedback loops allowed for tailored, site-directed facilitation. Critical juncture cases illustrate the complexity of EF and the need to often try multiple strategies in combination to facilitate implementation progress.

**Trial registration:**

The Protocol-guided Rapid Evaluation of Veterans Experiencing New Transient Neurological Symptoms (PREVENT) is a registered trial (NCT02769338), May 11, 2016—prospectively registered.

**Supplementary Information:**

The online version contains supplementary material available at 10.1186/s43058-021-00180-3.

Contributions to the literature

- Although there have been efforts to standardize the reporting of external facilitation activity, our results illustrate the importance of evaluating facilitator activity within the context it is being delivered and in relation to whether or not it was successful.
- The model of multi-tiered external facilitation described here demonstrates the crucial role that monitoring and analysis, from multiple vantage points, can have in informing and supporting tailored implementation support.
- Critical junctures illustrate how external facilitator activities can positively change the course of local implementation trajectories, especially during periods of uncertainty.

## Background

Facilitation is an interactive implementation strategy aimed at supporting problem-solving and interpersonal relationships [1]. Facilitators are responsible for helping drive and motivate change within an organization, and serve as a resource for implementing and tailoring change efforts [2]. Facilitation both externally and internally occurs in dynamic contexts, where political and cultural factors influence targeted change processes over time [3]. Facilitators often need to respond “in the moment” rather than in predetermined ways [4–6]. Facilitation is thus a relational practice [7–9]. Facilitators need to know when and how to intervene to be effective. They also require multiple, complex skills to guide people through change processes [10], using external benchmarks and understandings of local context.

Facilitator roles and activities are numerous [2, 11–13]; facilitators frequently engage in activities like assessing progress and audit and feedback [14] as part of a suite of strategies [2, 12, 15].

Given these wide ranging activities, previous work has illustrated the importance of models of external facilitation that go beyond a lone facilitator [5, 11, 16–18]. Researchers have described the roles and impacts of facilitator dyads, such as in blended external and internal facilitation designs [19–24], and mixed discipline facilitator designs [16–18]. These models involve sharing and coordinating different functions of facilitation, though manuscripts rarely detail how they work together. Lessard and colleagues [5] made the relational aspects of facilitation more apparent by extending the facilitation relationship beyond internal and external facilitators to include the research group, the organization, and other external change agents. They described how a research group, for example, provided ongoing support and feedback to external facilitators, and how the external facilitators in turn acted as a liaison for the research team with the implementation setting.

Studies have described what facilitators do; however, less is known about how contextual factors prompt facilitators to adopt certain roles at different times to different recipients within and across an organization seeking change [5, 20]. Outside of the blended external and internal facilitation model, other models of external facilitation remain underexplored in the published literature. This analysis builds on the work of Lessard and colleagues [5] to articulate a model of facilitation that included a facilitator dyad joined by other parts of the implementation team, and describes the role that relationships, and monitoring and feedback at all levels played in deploying tailored external facilitation to support site implementation.

## Methods

We sought to understand how external facilitation was employed as an implementation strategy and perceived by its stakeholders during a national quality improvement (QI) initiative to improve transient ischemic attack (TIA) care. We used case studies to examine how locally tailored external facilitation (EF) was activated during critical junctures. This project featured multilevel implementation facilitation, involving frequent feedback between site quality improvement teams and external facilitators, with behind the scenes support from an interdisciplinary national implementation support team.

### Design

The Protocol-guided Rapid Evaluation of Veterans Experiencing New Transient Neurological Symptoms (PREVENT) was a stepped-wedge trial to improve the quality of TIA care (NCT02769338) [25]. The implementation trial and its methodology was previously published. To summarize briefly, each of our three waves involved implementation at two VA facilities (*n*=6); each facility had a 1-year active implementation period. Sites were eligible to participate if they had measurable gaps in TIA quality of care and treated at least 10 eligible TIA patients a year. The Indiana University institutional review board and the Richard L. Roudebush Department of Veterans Affairs (VA) research and development committee approved the study. Clinical staff from six facilities volunteered to participate in PREVENT. Each site developed a local interdisciplinary QI team, led by self-selected local champions who stepped forward to support ongoing implementation of the project. The target of the intervention was the facility and how it provides TIA care, not individual patients. We have used the StaRI checklist to assure adherence to relevant reporting guidelines (see Additional file 1).

### Implementation strategies

PREVENT used EF as a key implementation strategy to tailor site support across the five intervention components (a quality of care reporting system, clinical programs, professional education, electronic health record tools, and quality improvement support). Sites participated in kickoff events to begin their 12-month active implementation period. Each month during active implementation, members of the local QI teams received TIA care quality data and participated in cross-site, virtual collaborative calls. A website (the “Hub”) provided access to data, tools, local project pages, and other resources. Facilitation across data, activities, and the Hub was principally provided by a QI nurse and QI physician, with support from the full national implementation support team. After active implementation, sites still had access to the tools, resources, and external facilitators; however, external facilitators ceased reaching out to sites for updates and data, and proactive problem-solving.

### Data collection

Data for this analysis is from the PREVENT mixed-methods evaluation: QI nurse and physician facilitator correspondence with sites, stakeholder interview transcripts, and collaborative call debrief transcripts.

The prospective QI nurse and physician facilitators’ emails and Skype chat logs with sites were saved and logged using a spreadsheet adapted from the Implementation Facilitation Training Manual [13]. Emails and chat logs were reviewed by members of the evaluation team to identify site-directed episodes of EF. Episodes were defined as cohesive interactions, often contained in one email chain but that could span several weeks and were related to a specific request (e.g., asked to share something on a collaborative call), problem (e.g., preemptive problem-solving), or question (e.g., clarification about a site activity). Episodes that only consisted of local-site-only activity (i.e., no EF) or cross-site activity (i.e., EF provided to all sites) were excluded from the analysis. The QI nurse or physician’s activity in each episode was independently scored for the presence or absence of each of the 12 EF activities in the EF log (e.g., goal setting, brainstorming solutions) by two implementation scientists (see Additional file 2). Scoring differences were reconciled through discussion.

Semi-structured stakeholder interviews were conducted in-person at 6 months and completion (12 months) of active implementation, and by phone during sustainability by trained implementation team members. Site champions and a purposive sample of others identified as engaged in the local QI effort were invited to participate. Interview questions focused on stakeholder experiences with PREVENT, including external facilitation. Each interview transcript was independently coded and then reconciled as needed by two trained project members using a codebook based on both a priori and inductive codes on the overarching implementation process guided by the Consolidated Framework for Implementation Research (CFIR) Implementation Process Domain [26] and PREVENT-specific factors and characteristics.

### Data analysis

We applied a hybrid thematic analysis approach [27] on the External Facilitation code report; themes were refined through discussion with the implementation team.

After each monthly collaborative call (*n*=22), QI facilitators and members of the national implementation support team met to debrief about progress at different sites. To understand site-specific EF patterns over time, transcripts of call debriefings were reviewed to identify where implementation support team members expressed concerns about site progress, how they problem-solved, where there was involvement from external facilitators, and where problem resolution occurred. Episodes were discussed with the implementation team and two case studies were selected for presentation in this manuscript to demonstrate the diverse ways EF was used.

## Results

### Participants

Forty-two unique individuals participated in a total of 78 interviews that occurred at 6 months (*n*=32), 12 months (*n*=27), and sustainment (*n*=19). Participants represented multiple different disciplines; across sites, the most common were neurology, emergency medicine, pharmacy, and nursing.

### General and site-directed activities by phase

As part of the PREVENT intervention components, feedback loops were built between the QI teams and the PREVENT national program (a new quality of care reporting system, quality improvement support and virtual collaborative delivered by a QI nurse) and among local QI teams (cultivating clinical programs and supporting local networking and care coordination). In delivering the PREVENT program, to support these efforts, there emerged multiple feedback loops within the national program team. In practice, this resulted in an elaboration of the planned QI nurse EF, to a multi-tiered EF system that involved site-facing support and extensive backstage work.

**Fig. 1:**
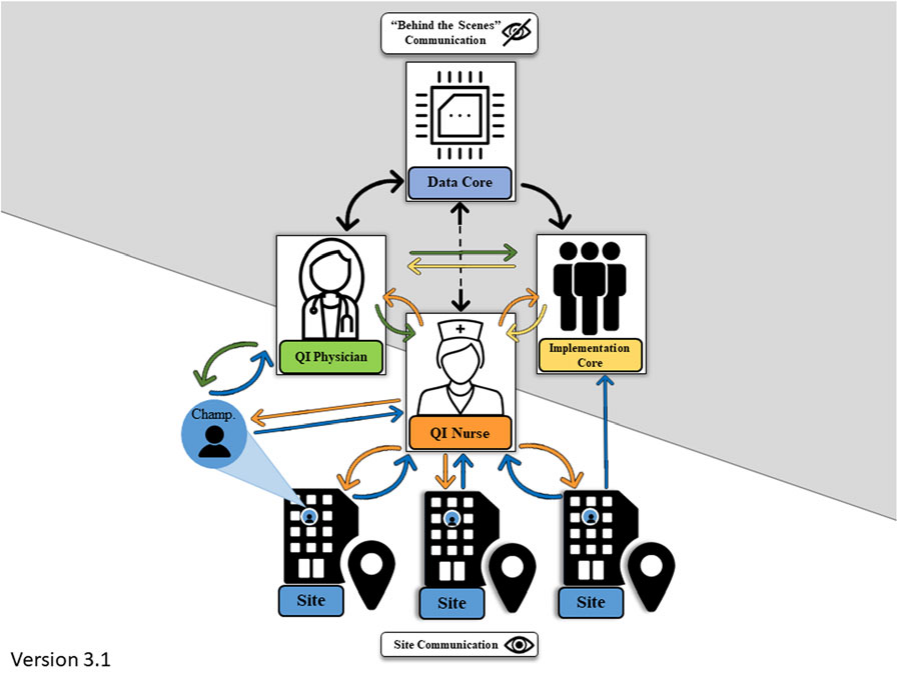
PREVENT multi-tiered external facilitation PREVENT multi-tiered facilitation. To support the delivery of the PREVENT intervention components (e.g., quality of care reporting system/audit and feedback, quality improvement support, clinical programs), feedback loops emerged between the mostly behind the scenes work by the Data and Implementation Cores and the site-facing work of the QI nurse and QI physician. This coordinated effort to monitor and make sense of site progress resulted in a multi-tiered EF system

The PREVENT multi-tiered EF involved a QI facilitator dyad and the national implementation support team (see Fig. 1). QI facilitators included a QI nurse and the project PI, an internal medicine physician (QI physician), both of whom provided site-directed support. The QI nurse was the primary point of contact for sites, facilitated site use of program components, assisted with problem solving, conducted outreach, provided logistic support, and offered encouragement and praise in coordination with and under the direction of the QI physician. Members of the interdisciplinary national implementation support team had expertise in data or implementation science (referred to as Data Core and Implementation Core), were responsible for ongoing evaluation of PREVENT, and provided comments and observations mostly related to the QI facilitator site-facing support. Groups met each week and after other activities with sites (e.g., monthly collaborative calls, periodic site visits) to discuss site progress and make decisions about and plan EF.

As a dyad, the QI nurse and QI physician served separately, though coordinated and sometimes overlapping EF roles (see Additional file 3). Both the QI nurse and QI physician built trust with local QI team members and encouraged and recognize participant success. Together they extensively planned facilitation activities which were usually delivered by the QI nurse.

During interviews, QI team members described site-facing problem-solving, feedback, and accountability EF support they received. The QI nurse helped participants understand how to effectively use PREVENT components (“Education”), particularly related to how they could use audit and feedback tools (e.g., the data Hub). She also helped sites problem-solve and, as needed, connected them to experts (“Networking”) to help them overcome challenges (e.g., implementing a clinical informatics tool). Many of the QI nurse’s interactions with sites (multiple per month) served to elicit site updates (“Process monitoring”) and included notifications about updated Hub data (“Audit and feedback”). Some participants described how this tracking and feedback kept them on track: “it’s not as fun to kind of necessarily maintain something. So you all kind of periodically nudging us is a good thing. … With the nudges come sort of the carrots as well of feedback in terms of how we’re actually doing. Putting them in context is very helpful” (QI team member, site 101, 12 months).

“Nudges” were feedback that helped them prioritize the project, situated the project in terms of practical performance metrics, and often prompted sites to action. For example, one champion said reminders “keeps you on your toes” (champion, site 104, sustainment). Requests for site updates (sometimes multiple) before collaborative calls encouraged reflection and action. Updates made the QI nurse a source of accountability, while also potentially a trusted source for problem solving:

I send [QI nurse] any progress that we make an on the order sets because I’m constantly worried that she feels like that we’re just going to stagnate... any time that I’ve had like a question about anything or if I wanted to like bounce an idea or ask for feedback, I have like no qualms about emailing [QI nurse] or hitting her up on [instant messaging]. (champion, site 103, 6 months)

#### Site-directed external facilitation activities and site implementation

In addition to the general EF activities delivered to all sites described above, the QI nurse and QI physician dyad also tailored support to each site. On average, sites received 24 episodes of site-directed facilitation prior to and during active implementation. The QI nurse was involved in many more site-directed facilitation episodes than the QI physician (see Table 1). Both engaged in preparation and planning with sites (e.g., for kickoffs or collaborative calls), promoted networking (e.g., encouraged people to get together), monitored processes (e.g., elicited information about site implementation for the Implementation Core), and provided education (e.g., sent scholarly citations) (see Additional file 4 for excerpts from communications). In her site-directed facilitation, the QI physician was more likely than the QI nurse to engage stakeholders (e.g., gain buy-in from site leadership), perform audit and feedback (e.g., give data from the Data Core), and brainstorm solutions (e.g., offer suggestions for local care gaps), and to do these things during pre-implementation. Across sites, 30% of the site-directed EF was initiated by site champions.

**Table 1:** Site-directed external facilitation (EF) by facilitator during pre-implementation and active implementation

**Site-directed EF episodes**				**Site-directed EF type**													
				Preparation and planning		Stakeholder engagement		Education		Process monitoring		Audit and feedback		Networking		Brainstorming solutions	
	Grand total	Mean per site	Percent pre-implementation	Total episodes	%^b^(Total episodes (TE)/grand total (GT)	Total episodes	% TE/GT	Total episodes	% TE/GT	Total episodes	% TE/GT	Total episodes	% TE/GT	Total episodes	% TE/GT	Total episodes	% TE/GT
QI nurse	150^a^	25	7%	74	49%	5	3%	55	37%	65	43%	26	17%	71	47%	26	17%
QI physician	14^a^	2.3	38%	1	7%	6	43%	8	57%	5	36%	7	50%	4	29%	5	36%
Sites	Grand total		Percent initiated by champion	Total episodes	% (total episodes (TE)/grand total (GT)	Total episodes	% TE/GT	Total episodes	% (TE/GT)	Total episodes	% (TE/GT)	Total episodes	% (TE/GT)	Total Episodes	% (TE/GT)	Total episodes	% TE/GT
101	22		23%	10	46%	0	0	11	50%	10	46%	3	14%	9	41%	4	18%
102	30		30%	18	60%	5	17%	9	30%	12	40%	5	17%	13	43%	4	13%
103	39		54%	19	49%	1	3%	12	31%	19	49%	13	33%	20	51%	12	31%
104	21		0%	8	38%	1	5%	7	33%	10	48%	0	0	8	38%	2	10%
105	22		41%	9	41%	1	5%	11	50%	8	36%	5	23%	13	59%	3	14%
106	26		7%	11	42%	3	12%	10	38%	11	42%	6	23%	12	46%	6	23%
Median (across sites)	24		30%	10.5	44%	1	5%	11	36%	11	44%	5	20%	13	45%	4	16%

^a^Four episodes involved both QI nurse and QI physician external facilitation. Those episodes have been counted for both the QI nurse and the QI physician

^b^Percentage of total site-directed episodes that included that type of facilitation activity (total episodes of that EF type/grand total site-directed EF episodes by EF and by site). EF episodes could involve more than one type of facilitation (e.g., an email that provided updated data [Audit and Feedback] and ideas about how to address deficiencies [Brainstorming Solutions]) so the total percentage across facilitation types will be greater than 100%

Site-directed EF varied in quantity and type, though overall networking (in 45% of the site-directed EF episodes) and preparation and planning (44%) were the supports most often provided (Table 1). Site 103 was at the highest end of most metric ranges: they had the most site-directed EF episodes [28], their champion initiated 54% of their episodes, and they had the highest percentage of episodes that included process monitoring (49%), audit and feedback (33%), and brainstorming solutions (31%). Site 104 had the fewest number of site-directed EF episodes [21], no contacts initiated by the site champion, and had the lowest percentage of episodes that included preparation and planning (38%), networking (38%), brainstorming solutions (10%), and audit and feedback (0%).

**Fig. 2:**
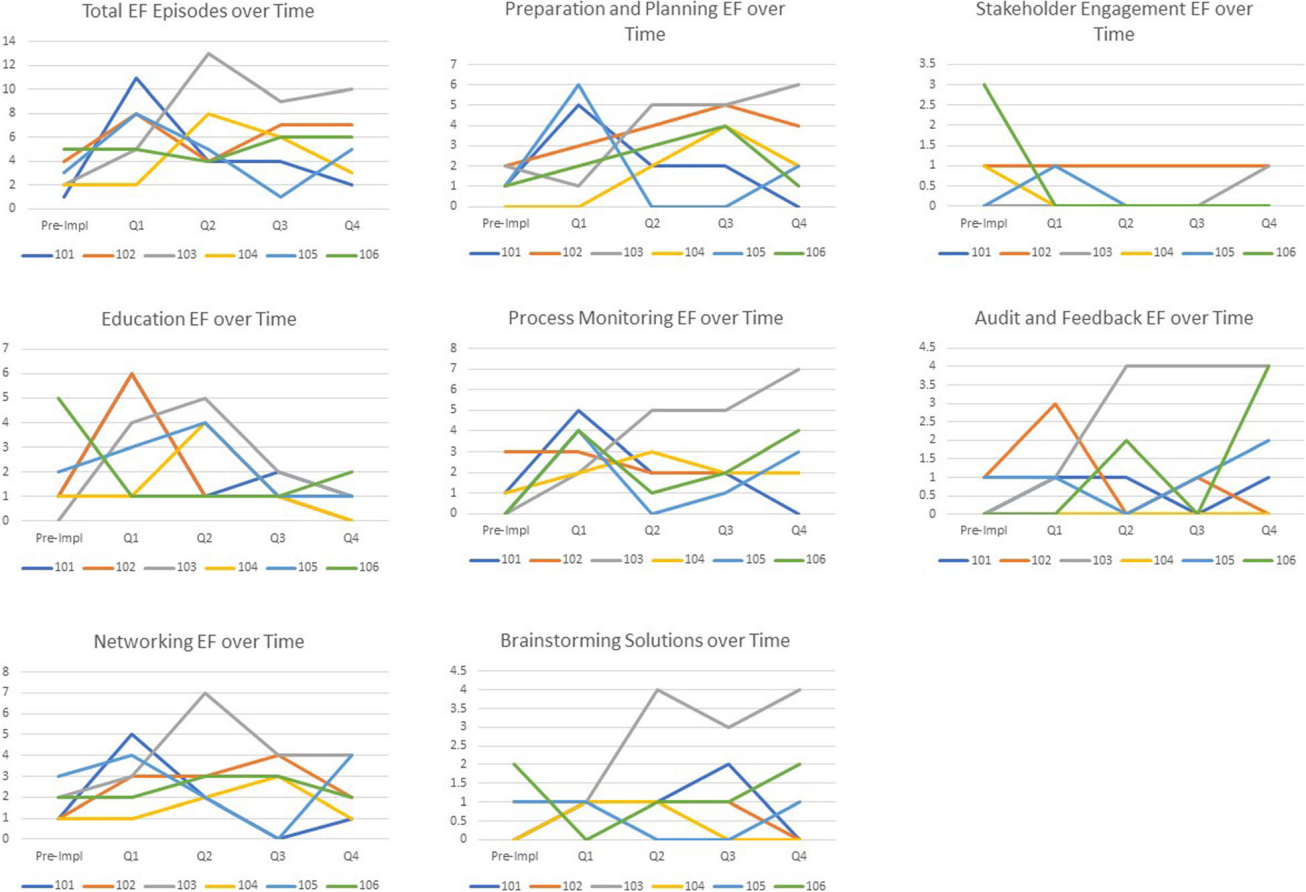
Site-directed facilitation activities by time period (pre-implementation and active implementation quarter) Panel of PREVENT site-directed EF by activity type over time

There was also variability in EF activity within sites over time (Fig. 2). Sites varied in terms of when they received more or less site-directed EF. In some cases, more EF corresponded to increased site implementation activity (e.g., to support use of data to direct activities), in other cases it occurred during site inactivity (e.g., to suggest solutions to challenges). Across sites, there was a temporal trend for fewer site-directed education EF across the active implementation period. Facilitators often spent more time during early quarters clarifying and explaining the PREVENT program and helping sites develop local education materials.

### Critical EF junctures

To better understand the varied patterns in site-specific EF activities over time, we looked across our data sources to examine what was happening at the sites when EF was activated. Through constant monitoring of feedback loops, the PREVENT facilitation team surveilled for potential implementation problems and discussed during team meetings potential interventions. For two sites, concerns were raised repeatedly and led to interventions by the facilitators at “critical junctures” [17, 29]. These are times of tension and uncertainty about the project status (e.g., questions about goals and direction, if and how the project can move past a barrier), where decisions and actions by individuals can lead to changes in implementation trajectory.

**Fig. 3:**
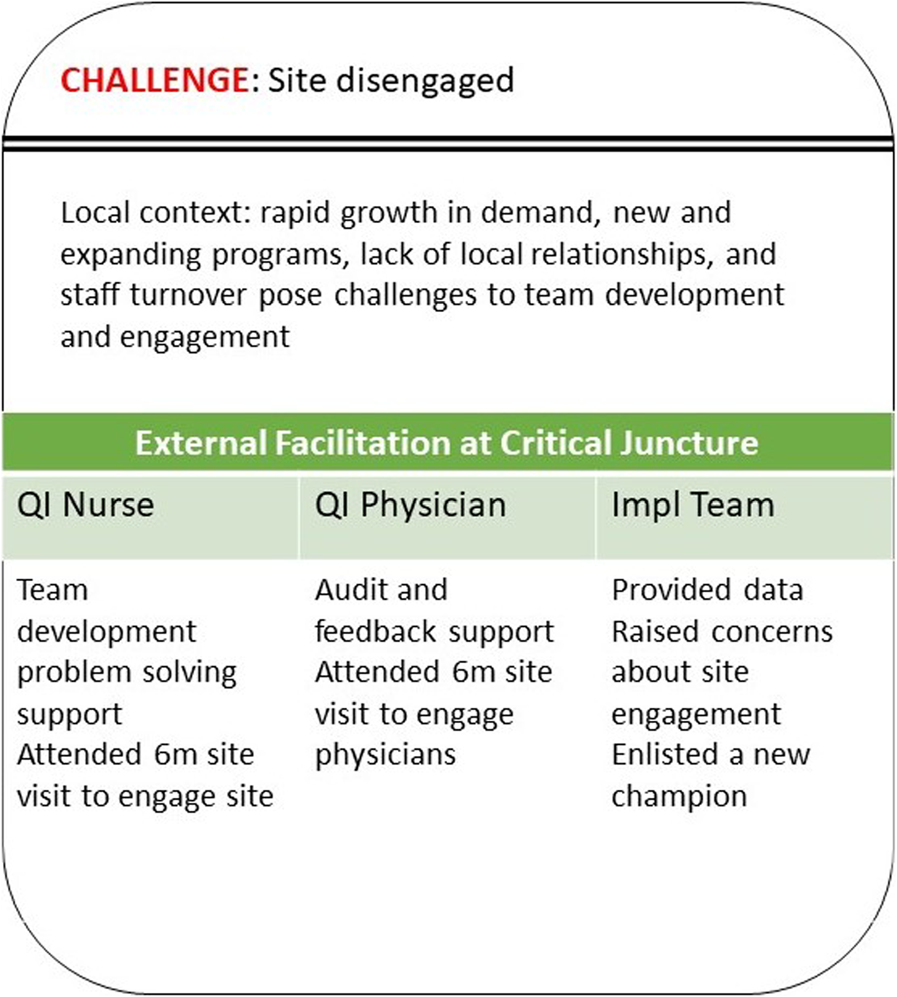
Site 106 critical juncture Summary of the context in which site 106’s critical juncture occurred and the EF responses by the different members of the PREVENT multi-tiered EF system

Site 106 struggled from the start to implement QI activities (Fig. 3). With a rapidly growing patient population, new and expanding programs, and staff turnover, local pressures made it difficult to convene a committed, local team. During the first 6 months, the QI facilitators and the national implementation support team reflected on and expressed concern about (during internal team briefings) lack of site engagement in the collaborative calls, team development, and implementation progress, and the loss of one of the co-champions. To assist in team building, the QI nurse reached out to the remaining champion to suggest potential local roles who could help. To prompt planning and action, using data provided by the Data Core, the QI physician sent an email that outlined the local site’s data, where that facility was doing well, and processes that could be improved. Prior to the 6-month evaluation team visit, a member of the Implementation Core strongly recommended that the visit be used as an intervention to re-engage the site. The QI physician traveled to the facility in person to engage local physicians throughout the site visit. While on site, a member of the Implementation Core enlisted an additional brand-new local champion. In the following months, the new champion was able to quickly engage the participation of another service, began participating in the broader PREVENT community of practice, and took responsibility for working and monitoring progress toward goals. However, the new champion continued to be challenged to engage the needed stakeholders and to understand how to implement PREVENT fully within the constraints of local context.

**Fig. 4.**
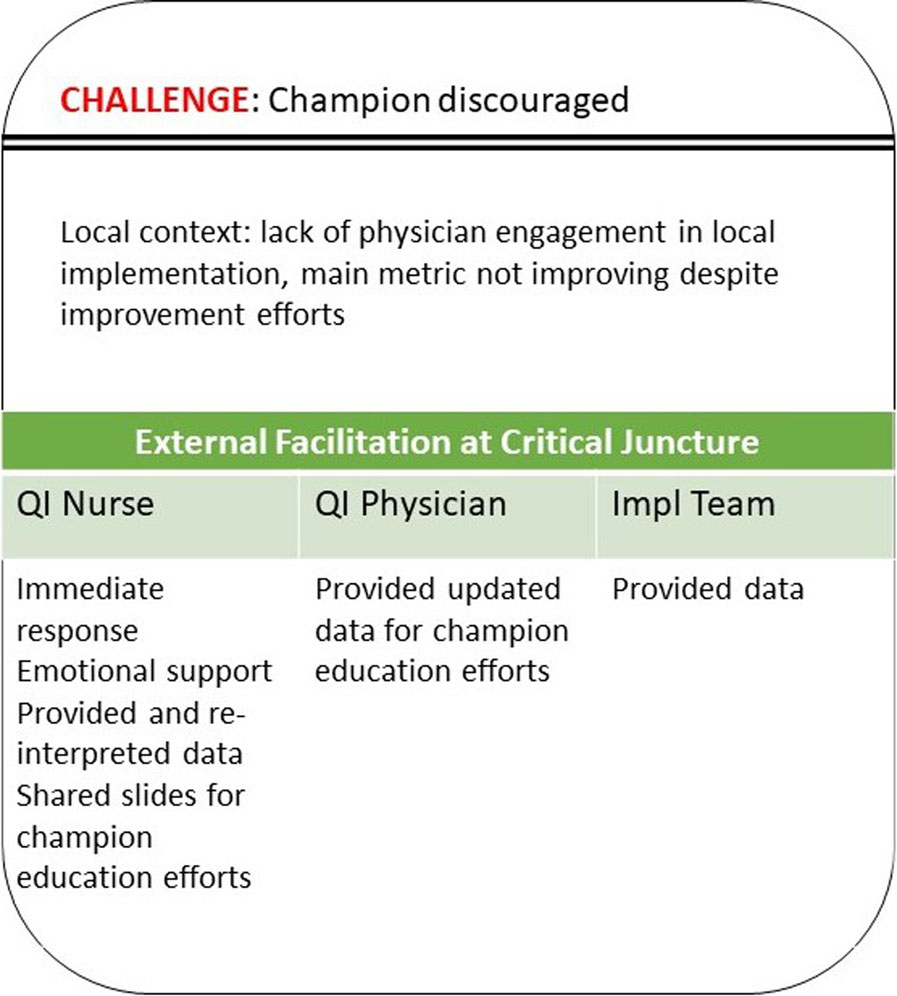
Site 103 critical juncture Summary of the context in which site 103’s critical juncture occurred and the EF responses by the different members of the PREVENT multi-tiered EF system

Site 103’s champion was engaged and highly motivated from the start of implementation (Fig. 4). Team debriefings frequently highlighted the implementation support team and QI facilitators’ confidence in the champion’s ability to problem solve challenges and praised her progress, even when noting the lack of local neurology engagement. Early into implementation, the QI nurse offered to invite some of the non-engaged stakeholders to the collaborative call, but the champion did not respond. Nine months into active implementation, the champion emailed the QI nurse discouraged by continued poor process of care metrics. The QI nurse immediately called the site champion to provide encouragement and a positive perspective on the process of care data. Over the next few days, through a series of instant messages and emails, the QI nurse provided patient-level data and helped to re-interpret the data to emphasize improvements in several processes of care and near misses with neurology consultations. The champion quickly responded by re-educating medicine attendings and talking with the neurology chief. With updated data from the Data Core that was provided by the QI physician, the champion made a presentation to the neurology service which helped convince the neurology service to agree to a critical process change. Although the champion continued to struggle with physician engagement throughout the sustainment period, during the 12-month and sustainment interviews she described this EF intervention involving data reframing and offering moral support as critical to enabling her to act:

I just felt like that I was working so hard, and I was like trying to cover all of these holes… and our compliance rate just ended up being like zero for the month … I really just wanted to quit because I just was really frustrated. … [QI nurse] was really helpful in allowing me to just kind of call and vent, and then she was also really very encouraging at saying like I know that your without fail rate is zero, but you also have to look at the improvements that you did make this month. … that was an interesting lesson to learn that you might feel like that you’re unsuccessful because of that one particular metric, but you have to look at the bigger picture… I took the weekend and came back and felt like okay, let’s tighten up and figure out what we need to address and move forward, and then the next month was better. (champion, site 103, 12 months)

### Sustainment

At the end of active implementation, without the structure of the PREVENT program (e.g., monthly calls) and the reminders from the QI nurse which prompted teams to communicate and act, many local teams stopped having regular check ins. A champion described the impact of losing external facilitation: “basically the net effect was that the group as a system for checks wasn’t as…. You know. Wasn’t as involved anymore. Right. So then that just or it just kind of went to the individual members to see what their responsibilities were.” (champion, site 104, sustainment) The loss of check-in emails led to sharp declines in team activity and coordination.

Another champion said that the loss of external facilitation during sustainment resulted in less motivation to and information that would help them innovate:

it’s not like we’re pushing for that innovation, like we’re not getting those ideas to think about to push to get to the next level. So I see that as a deterrent. I mean of course I could research it and learn more about it, and spend a lot of time, but I don’t have a lot of time to do that. So it was good to kind of just get that information pushed to us. (champion, site 106, sustainment)

However, not all sites felt the loss in the same way. Participants at site 101 said that because of the loss of external facilitation and integration of PREVENT into their existing stroke program, PREVENT was less a focus and data sharing among the group was diminished. However, the program was sustained in routine practice: “we’ve continued to, I guess, you say maintain even without the additional support or the constant reminders. Once we got the program situated, seems to be doing pretty well.” (QI team member, site 101, sustainment)

## Discussion

This study provides an application of multi-tiered EF where EF was a process to which multiple role contributed [11], and the implementation strategies were locally tailored based on context and often combined to overcome site critical junctures. Multi-tiered facilitation emerged during active implementation as a response to planned audit and feedback activities and site implementation progress; it provided a flexible and productive implementation strategy within a larger bundled set of interrelated strategies in this study. We uniquely show how tailored, site-directed QI nurse facilitation was supported by the QI physician and backstage work of the broader national implementation support team. Site-directed activity counts helped us understand general differences in EF “touches” across sites and within sites over time, but it was difficult to interpret their significance without understanding what they specifically involved and how they responded to site-specific challenges. Consistent with other studies, we found there was not a one-sized fits all approach to EF [30] and that facilitation was a non-linear process [4, 5, 31]. We additionally show how the implementation team’s sensemaking helped determine EF deployment over time.

Through their activities, facilitators flexibly used multiple implementation strategies [1], including audit and feedback, use of data experts, preparing champions, promoting network weaving, distributing educational materials, and holding educational meetings. Keys for QI facilitation in PREVENT were establishing trust and relationships (especially with site leads/champions), being approachable and responsive, providing enthusiastic encouragement and praise, and ongoing monitoring and team sensemaking to identify opportunities to intervene and recalibrate site trajectory. Sensemaking is a meaning making process whereby groups interpret situations and identify possible solutions for action [32, 33]. It was not an organizing principle for our work but emerged in this analysis as an important feature of the PREVENT multi-tiered external facilitation. Our data suggest that knowing when and how to intervene, as well as when and how to ask for help, was not always clear. Having multiple, sometimes overlapping efforts to “monitor the pulse” of local activities and built-in feedback loops were important for activating EF. Efforts to develop rapport with and closely monitor sites, from multiple directions, facilitated that feedback. While monitoring progress and follow-up are often a core and influential facilitator role [14, 30, 34], our work deepens our understanding of how that works to inform tailored EF particularly at points of implementation bottlenecks. Building in time for the PREVENT team to cultivate relationships, systematically reflect on and evaluate feedback, and plan together was critical for sensemaking and mobilizing facilitation support at critical junctures. As Simpson et al. [35] and Lanham et al. [36] advised, setting aside time for the group conversation was foundational to this process. The built-in feedback loops and tiered reflecting and evaluating that were planned into this study provide an example of a learning health system in practice [37].

The critical juncture cases illustrate the complexity and potential impact of EF. Both cases involved the external facilitator trying to enhance the capacity of local QI teams and providing data to support local activation and problem-solving, yet not all interventions had their intended impacts. The local QI team often had to trial several different locally tailored strategies until they found something that worked. Future work should follow Moussa and colleagues’ lead to record barriers to which facilitator activities are directed and whether the strategy worked [38], in addition to what activities or strategies they deploy. Our cases focus on specific EF episodes that helped keep sites engaged and moving primarily through a local champion. However, both sites continued to experience challenges and additional site-directed EF activities occurred after the critical junctures. The two cases also had very different site-directed EF patterns, although both struggled with local interdisciplinary engagement. Site 103 had many more “touches” and a champion who reached out to the QI nurse very frequently. Site 106 had many fewer site-directed episodes and fewer champion reach outs, even after the new champion was engaged. These differences likely reflect the different skillsets of the champions as well as their relationships with local stakeholders and the QI nurse. Just as project fit and alignment with champions or internal facilitators is important to the success of the facilitation strategy [21, 39], so too may be the nature and quality of relationship between the internal and external facilitator to the success of the facilitation strategy [7, 20, 28].

QI nurse activities often involved administrative and logistic support (preparation and planning). Each month, sites received multiple group emails requesting site updates for the collaborative call, providing information about the collaborative calls (e.g., agendas, how to receive continuing medical education credit), and regarding new data on the Hub. Though mundane and to our knowledge not described in depth in the facilitator literature, these activities helped maintain engagement, kept the project top of mind, and ensured that technical difficulties were not a barrier to site progress. They also served strategic implementation purposes for monitoring (e.g., directly and indirectly eliciting information about site progress) and accountability. As reflected in sustainment interviews, we learned that in addition to the importance of general capacity building and skill transmission [2, 40, 41], a challenge for sustaining programs like PREVENT is building in specific local capabilities for ongoing monitoring and accountability.

Experiences from this analysis can inform ongoing methodology work to document and understand the role of EF. Our EF log did not adequately reflect the degree to which QI facilitators gathered and processed data for sites, or the meaningful ways they celebrated and appreciated site achievement. Data support was a valued aspect of EF. Despite the intended user-friendly design of the data Hub, individuals still struggled with both data gathering and interpretation. Hub office hours included examination of and sensemaking about performance. The 103 case illustrated the mutability of the data and the ways that the facilitators were able to reframe how participants understood and acted upon data, and thus influenced local sensemaking. The case also demonstrates the positive perspective that EF facilitators brought to discussions of site progress. In emails and collaborative calls, facilitators consistently congratulated and highlighted site successes; behind the scenes, they similarly expressed appreciation of site positive gains. It is unclear how effective sites would have been in implementing PREVENT had they not had this reinforcing, external support. These findings resonate with Berta and colleagues’ proposal that facilitation has the potential to effect higher order learning and organizational change through multiple micro-processes and activities, like introducing new ideas and encouraging change, and meta-routines like reflecting on and updating their understandings [40].

Although the large national implementation support team was beneficial for monitoring and tailoring support, and provided a wider reach into local QI teams compared to a more centralized facilitation approach [42], as others have noted [43], facilitation is resource intensive. Following the Ritchie et al. model for facilitation skill transmission [41], we could have had a process for transferring key tasks to sites (e.g., have sites take ownership of collaborative calls, encourage them to collect their own QI data, help QI teams establish local bodies for accountability) or automated certain functions (e.g., reminders to update goals). This may have benefited sites for the long term, however champions and QI teams had limited, if any, dedicated time and local support for PREVENT (e.g., systems redesign office help, data support systems). Similarly, while facilitators addressed immediate barriers to local PREVENT implementation, larger structural and cultural issues (e.g., difficulty engaging physicians because of policies around physician effort and clinical focus) that were challenges to implementation and sustainment often could not be practically addressed within a limited, voluntary program. This leaves unresolved the issue of to what extent EF should be engaged to address locally specific systemic issues that could be worked around for short-term implementation but nonetheless still pose a risk to long-term sustainability.

This study had several limitations. We were unable to tease out the specific impacts of EF on site success due to the complex interaction between our implementation strategies. EF in the VA may work differently than in other, non-integrated healthcare systems. Our analysis focused on the presence of EF activities and not their absence and hence did not identify the points in time where external facilitators did not act, either based on a decision to not intervene or a failure to recognize a possible point of intervention.

## Conclusion

PREVENT’s multi-tiered facilitation made use of emergent, built-in feedback loops between the QI facilitators and the national implementation support team to identify places for and tailor EF. Using critical junctures, we were able to show how the groups worked together to problem solve site challenges and direct, and redirect, EF support.

## Supplementary Information

**Additional file 1.** StaRI checklist for implementation science standards for reporting met in this manuscript.

**Additional file 2.** External Facilitation Activity Types and Definitions. Table displaying each facilitation activity type and the definition used to assess site-directed EF communications.

**Additional file 3.** Multilevel External Facilitation Roles and Across-Site Activities. Describes in more detail the specific roles and activities of the QI facilitators, Implementation Core, and Data Core. Two figures display the differing and combined roles, as well as the dual external facilitation provided by the QI nurse and QI physician across all sites.

**Additional file 4.** Examples of Site-Directed External Facilitation. Table with expanded excerpts from site-directed EF communication, corresponding to the types of EF activity for which it was coded.

## Data Availability

The datasets generated and/or analyzed during the current study are not publicly available due to concerns about participant privacy. De-identified facilitator logs with facilitation activity scoring are available from the corresponding author on reasonable request.

## Abbreviations

EF: External facilitation
PREVENT: Protocol-guided Rapid Evaluation of Veterans Experiencing New Transient Neurological Symptoms
QI: Quality improvement
TIA: Transient ischemic attack
VA: Veterans Health Administration
