# Supplementary material for: Multi-tiered external facilitation: the role of feedback loops and tailored interventions in supporting change in a stepped-wedge implementation trial
Source: Implement Sci Commun. 2021 Jul 27;2:82. doi: 10.1186/s43058-021-00180-3 (PMC8317410; doi:10.1186/s43058-021-00180-3)
Supplement: Supplementary file 2 — Additional file 2. External Facilitation Activity Types and Definitions. Table displaying each facilitation activity type and the definition used to assess site-directed EF communications. [file 43058_2021_180_MOESM2_ESM.docx]

**Additional File 2. External Facilitation Activity Types and Definitions**

| Facilitation Activity | Definition | Examples |
| --- | --- | --- |
| Preparation and planning | Help sites identify local change agents or champions, facilitate selection of program components that will best meet local needs, prepare and plan for monthly Collaborative Calls (CC) by soliciting sites’ updates and new goals | Identifying names of participants to invite to CCs, requesting/ confirming information to be presented on CC, requesting participants to present on CC |
| Stakeholder engagement | Engaging facility leadership directly through product and service line meeting presentations about the PREVENT program. Helping champions prepare to engage leadership | Inviting local leaders to Kickoffs and end of active implementation graduation ceremonies. |
| Education | Providing information about program and previous outcomes to leadership to ensure they are aware and supportive of program, educating clinical personnel on program components, how to access or use them, providing champions with educational tools to facilitate implementation, and educating participants on interpretation of performance feedback reports. | Educational materials provided during Kickoffs and CCs, responses to requests for information, directing sites to the online Hub for tools and materials, and providing explanations for how to post materials to the Hub or to sign up for continuing medical education credit. |
| Ongoing process/Quality monitoring | Reviewing implementation process outcomes | CC goal updates, and discussions with or evaluative comments made to site champion about their performance. |
| Program adaptation | Working with site participants on adaptations of the program to fit local context and needs, reviewing adaptations during collaborative calls/emails/individual calls | Assisting with site initiatives (e. g., ED TIA protocol, TIA Education brochure), and linking site to experts to adapt electronic health record tools to their own system. |
| Data audit and feedback | Accessing data through the PREVENT online Hub, reflecting and evaluating on the impact of efforts on the main outcome measures, and clarifying questions about data. | Questions about metrics and what they included, measure specifications, and responding to requests for patient level data. |
| Program marketing/ development | Supporting marketing activities of the PREVENT program across VA, nationally, regionally and local levels, identifying marketing opportunities and strategies through communication (calls, emails) with participants. | Participant presentation of improvement activities during CC, sharing site protocol/template on PREVENT Hub, presenting local teams’ QI achievements which are then edited for local publications – Facility and regional newsletters. |
| Network Development | Working to develop a learning and networking collaborative among sites through Collaborative Calls and other opportunities, supporting participants’ use of the Virtual Collaborative in conjunction with their own experiences and encouraging and supporting networking within the collaborative for problem solving and education, using participants as educators themselves in regional and local efforts/trainings. | Bringing site’s clinical informatics staff in contact with Indianapolis clinical informatics expert to share Patient Identification Tool; Inviting site Pharmacists to present on medications related to the process improvements; EM physician presentation of their ED against medical advice protocol. Encouraging members to attend collaborative calls to share with and learn from others. |
| Identification of barriers | Assisting local teams to identify implementation barriers. | Kickoff discussions to identify improvement opportunities, and email/telephone contacts to discuss challenges. |
| Brainstorming solutions | Assisting local team in problem resolution. | Kickoff discussions regarding where and how to improve processes, CC updates involving conversations about overcoming challenges, and site-direct email/telephone support regarding specific problems. |
| Goal setting | Facilitating local teams’ setting and communicating goals at Kickoff event, through Collaborative Calls, or via email updates. | Facilitated Kickoff goal setting, having teams communicate updated monthly goals during CCs. |
| Other | Anything else not captured by the above categories. | Request for link for symptomatic carotid stenosis definition citation, request for representation to attend CC when champion unable to attend |
