# Supplementary material for: Multi-tiered external facilitation: the role of feedback loops and tailored interventions in supporting change in a stepped-wedge implementation trial
Source: Implement Sci Commun. 2021 Jul 27;2:82. doi: 10.1186/s43058-021-00180-3 (PMC8317410; doi:10.1186/s43058-021-00180-3)
Supplement: Supplementary file 3 — Additional file 3. Multilevel External Facilitation Roles and Across-Site Activities. Describes in more detail the specific roles and activities of the QI facilitators, Implementation Core, and Data Core. Two figures display the differing and combined roles, as well as the dual external facilitation provided by the QI nurse and QI physician across all sites. [file 43058_2021_180_MOESM3_ESM.docx]

**Additional File 3. Multilevel External Facilitation Roles and Across-Site Activities**

The Protocol-guided Rapid Evaluation of Veterans Experiencing New Transient Neurological Symptoms (PREVENT) was a quality improvement initiative. Details of the trial have been reported elsewhere (Bravata et al. 2019). The objective was to implement and evaluate a multi-component intervention to improve the care of Veterans with transient ischemic attack (TIA). The components addressed known gaps in TIA care: professional education, quality reporting system, clinical programs, electronic health record tools, and quality improvement support and virtual collaborative. The components were supported by a multi-tiered external facilitation team, herein referred to as the National Implementation Support Team (see Figure 1).

Figure 1. Roles and responsibilities of the PREVENT National Implementation Support Team
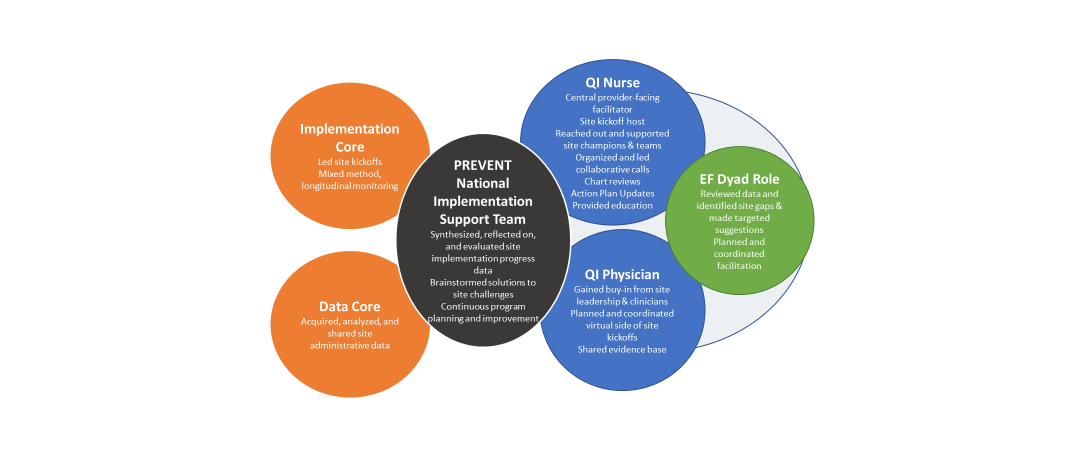


The QI physician and nurse held complementary, site-facing, EF roles (see Figure 2). The QI physician was the project’s clinical authority, engaged with site leadership and clinicians to create pre-implementation buy-in, suggested topics for collaborative calls, and provided medical and technical expertise support during site implementation. The QI nurse hosted kickoffs (e.g. provided food), consistently engaged and walked sites through project components (e.g., the data Hub, collaborative calls), and was the primary point of contact. As a dyad, they met weekly to review site data, discuss progress, plan for events, and discuss potential interventions to support site success.

Figure 2. Dual external facilitation by QI nurse and QI physician across major PREVENT tools


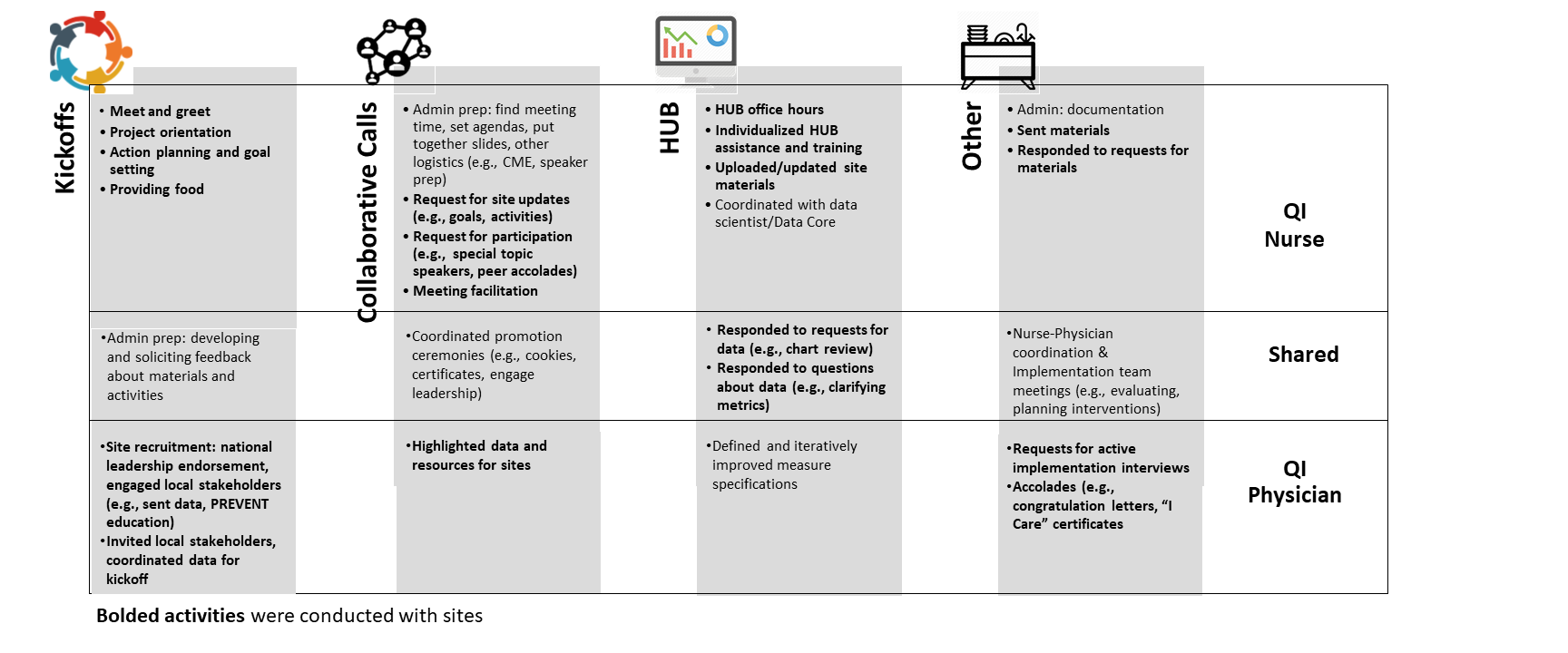


The EFs were supported mostly behind the scenes, by Data and Implementation Cores. The Data Core was primarily responsible for acquiring and reporting administrative data and helped educate site staff about metrics. The Implementation Core evaluated the implementation effort at both the national and site levels. The Implementation Core relied on data collected by the Data Core and QI nurse, as well as data independently collected through interviews with site participants and site visits.

These four groups or individuals made up the National Implementation Support Team. Each, to varying degrees, interacted with site participants, monitored site progress, and helped plan and implement program elements (e.g., Kickoffs, data Hub, Collaborative Calls). They reflected on and evaluated site projects based on their interactions with and observations of sites, during multiple weekly team (and sub team) meetings, monthly collaborative call debriefs, and periodic site visit debriefs. Questions raised about site activities were relayed by the QI nurse through email or instant message to the sites, or were added to site visit interviews. When concerns were raised about the pace or direction of site activity, they collectively solved potential interventions that were delivered by the EFs.
