# Supplementary material for: Multi-tiered external facilitation: the role of feedback loops and tailored interventions in supporting change in a stepped-wedge implementation trial
Source: Implement Sci Commun. 2021 Jul 27;2:82. doi: 10.1186/s43058-021-00180-3 (PMC8317410; doi:10.1186/s43058-021-00180-3)
Supplement: Supplementary file 4 — Additional file 4. Examples of Site-Directed External Facilitation. Table with expanded excerpts from site-directed EF communication, corresponding to the types of EF activity for which it was coded. [file 43058_2021_180_MOESM4_ESM.docx]

**Additional File 4. Examples of Site-Directed External Facilitation**

| Description of Interaction | Excerpts from External Facilitator Emails | Type(s) of Facilitation Employed |
| --- | --- | --- |
| Engaging leadership to gain permission for site participation in PREVENT | *Site leadership declined site kickoff visit after the site had already expressed interest in participating in PREVENT. Within a day, the QI physician followed up to clarify the project. This was followed by multiple emails to potential site champions to consider how to potentially engage with leadership and gain their buy-in.*  QI physician: “I apologize that my initial email did not provide sufficient information about the PREVENT project.  I am writing to address the issues raised in the email string below and to ask you to reconsider your decision about our visit to the [Site name]:  This is a VA HSRD funded project with support from VA Patient Care Services (see attached letter from Dr. [name], Neurology and Dr. [name], ED).  Our team is comprised of VA employees; from the Indianapolis VAMC.  We are seeking to improve care and outcomes for Veterans with TIA and minor stroke.   - Veterans with TIA and minor stroke are at high risk of recurrent vascular events (e.g., stroke/MI/death) in the 90-days after the index event.  Data from non-VA sources demonstrates a dramatic 70% reduction in recurrent vascular events for patients who receive timely guideline concordant care.  Our goal is to reduce recurrent vascular events by improving timeliness of care for Veterans with TIA and minor stroke. - I have attached a presentation I gave to the national VA Stroke Quality Improvement Network (SQUINT) group earlier this month.  [highlights specific slide numbers and data]   I have attached a report providing [site name] specific quality of care data.  These data are provided only for your own quality improvement work. They are not monitored by any national VA entity.   - [detailed site-specific data]   As part of the PREVENT project, we will provide assistance and tools (e.g., nursing templates, CAC support to build locally-tailored data reports, access to a library of shared resources, etc…) to sites interested in improving care and outcome for Veterans with TIA and minor stroke. Please see the attached PREVENT information sheet that describes the project in more detail.  The purpose of the in-person interviews is to understand how care is currently being delivered at the [site name] and to ask staff how the PREVENT program materials can be refined to meet their needs.  My thanks for your consideration.  Regards” | Stakeholder Engagement  Ongoing Process Monitoring  Data Audit and Feedback |
| Targeted follow up after collaborative call | QI nurse: “Hi [name], Hope you are well. On the January call you mentioned working on a statin algorithm. I just wanted to offer assistance if we could provide any resources to you for that effort. Of course, you are aware of the Library on the Hub [Link] where most resources are located (the [site 1] ED TIA protocol and the Pharmacy program come to mind). But if we could help in any way, please don’t hesitate to ask. Your input on the calls and your work to improve TIA care for the Veterans at [site name] VAMC are very much appreciated! Thanks” | Education  Ongoing Process Monitoring  Networking  Brainstorming Solutions |
| Reassurance and encouragement when not meeting goals | *In response to a request to submit new goals, site Champion apologizes for not accomplishing anything in the prior month and expresses the need to set achievable goals this time.*  QI nurse: “Hi [name]  No worries and please don’t be too hard on yourself. I think it’s taken many of us some time to get back in the groove after the holidays – and then there’s the dreary winter weather. But spring is around the corner, more energy, and new goals to motivate 😊. The March call topic is Planning, Goal Setting, and Feedback – and here’s a preview – we’d like to re-look at the Kick-off Action plans. So maybe that is a place to look for a new goal? Talk soon” | Preparation and Planning  Goal Setting  Identification of Barriers  Brainstorming Solutions |
| Linking to others who can help, Encouragement, reframing | *Champion contacts QI nurse about being in a “funk” over the site’s “terrible” performance in recent monthly data update. QI nurse immediately calls to discuss the data and site efforts, and follows up with the following email.*  QI nurse: “I will follow-up on our discussion just now and send you the [data] by Thursday. Also, I’ll ask [QI MD name] and our stroke neurologist, [name], for suggestions regarding provider issues. You have initiated several terrific quality improvement activities at [facility name] and there are metrics that are showing that. I always think if there is 1 Veteran in whom a stroke is prevented, that is worth all the effort. Please be encouraged, call anytime, and let me know if there is any way we can be supportive of you” | Ongoing Process Monitoring  Data Audit and Feedback  Networking  Identification of Barriers  Brainstorming Solutions |
| Helping navigate metrics to use to make sense of local issues | *A champion contacts QI nurse as she is trying to troubleshoot why they have so many neurology consult fails. She reports her neurologists say they see 100% of TIA patients. Champion asks QI nurse how neurology consult administrative data is drawn.*  QI nurse: “That is a great question! I have copied below the numerator, denominator, and exclusions for the Neurology Consult process of care from our Measure Specifications (these are in the Library on the PREVENT IOP Hub should you want to view them for other processes of care also). From the chart reviews I sent you for December, it appears there was one patient for which there was a phone conversation with a Neurologist, but no documentation in the chart. For the other patient, there was no mention of Neurology and no documentation of a consult.  You can drill down on this metric via the Scorecard from your PREVENT IOP Hub Project Plan by Neurology consult for ED only vs. Admitted. From June through December 2018, depending on the month, only 33-89% of the admitted patients received a neurology consult, while for those patients who were discharged home from the ED, generally 100% had a Neurology consult documented, though in June the pass rate was 50% and it was 0 in July (1 ED patient). You could also add the drill down by Weekday/ Weekend if you think that might be helpful.” | Education  Data Audit and Feedback  Identification of Barriers |
